# Prevalence of HIV, Syphilis, and Other Sexually Transmitted Infections among MSM from Three Cities in Panama

**DOI:** 10.1007/s11524-014-9885-4

**Published:** 2014-06-14

**Authors:** Shilpa Hakre, Griselda B. Arteaga, Aurelio E. Núñez, Nelson Arambu, Bulbulgul Aumakhan, Michelle Liu, Sheila A. Peel, Juan M. Pascale, Paul T. Scott, The Panama HIV EPI Group

**Affiliations:** 1 United States Military HIV Research Program, Henry M. Jackson Foundation for the Advancement of Military Medicine, Bethesda, MD USA; 2 Instituto Conmemorativo Gorgas de Estudios de la Salud, Panama, Panama; 3 School of Medicine, University of Panama, Panama, Panama; 4 National HIV/AIDS and STI Control Program, Ministry of Health, Panama, Panama; 5 Del Valle University of Guatemala, Guatemala, Guatemala; 6 National Center for Communicable Diseases, Ministry of Health, Mongolia, Mongolia; 7 HIV Diagnostics and Reference Laboratory, United States Military HIV Research Program, Walter Reed Army Institute of Research, Rockville, MD USA; 8 Department of Epidemiology and Threat Assessment, United States Military HIV Research Program, Walter Reed Army Institute of Research, Rockville, MD USA; 9 Epidemiology and Threat Assessment, United States Military HIV Research Program, 6720-A Rockledge Drive, Suite 400, Bethesda, MD 20817 USA

**Keywords:** HIV, Syphilis, Sexually transmitted diseases, Sexual behavior, Respondent-driven sampling, Sampling hidden populations, Panama

## Abstract

Respondent-driven sampling (RDS) was used to conduct a biobehavioral survey among men who have sex with men (MSM) in three cities in the Republic of Panama. We estimated the prevalence of HIV, syphilis, and other sexually transmitted infections (STIs), sociodemographic characteristics, and sexual risk behaviors. Among 603 MSM recruited, RDS-adjusted seroprevalences (95 % confidence intervals) were: *HIV*—David 6.6 % (2.2–11.4 %), Panama 29.4 % (19.7–39.7 %), and Colon 32.6 % (18.0–47.8 %); *active syphilis*—David 16.0 % (8.9–24.2 %), Panama 24.7 % (16.7–32.9 %), Colon 31.6 % (14.8–47.5 %); *resolved HBV infection*—David 10.0 % (4.8–16.8 %), Panama 29.4 % (20.0–38.3 %), and Colon 40.6 % (21.9–54.4 %); *herpes simplex virus type 2*—David 38.4 % (27.9–48.9 %), Panama 62.6 % (52.8–71.0 %), and Colon 72.9 % (57.4–85.8 %). At least a third of MSM in each city self-identified as heterosexual or bisexual. HIV prevalence is concentrated among MSM. Preventive interventions should focus on increasing HIV and syphilis testing, and increasing promotion of condom awareness and use.

## Introduction

Panama has a population of 3,405,813 people^1^ and since the first HIV diagnosis in 1984, has had 12,313 cases of HIV. The country has the third highest HIV prevalence in Central America, which is estimated at 0.8 % in the general population,^2^ a male/female ratio of 3:1, and a death rate of 67.1 %. The main mode of transmission reported is through heterosexual contact followed by homosexual and bisexual. However, for a third of the cases, the mode of transmission is unknown.^3^^,^ 4

In Panama and Central America, a situation analysis of stigma and discrimination related to homophobia, transphobia (prejudice expressed against transgender people), the sex trade, and people living with HIV, reported that health centers are still places of discrimination.^5^ A group of experts from the Americas who met in 2009 to address the health promotion and health care needs of men who have sex with men (MSM) reported that the MSM population postponed clinical attention for extensive periods of time or chose not to disclose their sexual orientation in previous visits to health care facilities. Such barriers to care make categorizing the type of transmission that affects the country difficult.

Research shows that MSM are at higher risk of contracting HIV than the general population, even in countries with generalized epidemics. Most data on the relative contribution of MSM to the HIV epidemic as a whole has been generated in high-income countries, including the United States, Australia, and Western European countries.^6^^,^ 7 In Central America, HIV prevalence among MSM range from approximately 6 % in Honduras to 17 % in Mexico which is similar to HIV prevalence in South America which range from 9 % in Uruguay to 20 % in Chile.^8^ The last sero-survey among MSM in Panama, conducted more than 10 years ago, reported an HIV prevalence of 8.9 % and low prevalence of other sexually transmitted infections (STIs).^9^ Current HIV and other STI prevalence estimates among MSM subgroups are unknown. According to UNAIDS/WHO, surveillance studies can provide valuable information for the design of specific interventions where HIV is concentrated in subgroups that have high-risk behavior,^10^ but these interventions should be carried out according to the sociocultural characteristics of each group. We conducted a survey to estimate the prevalence of HIV and other STIs, and high risk behaviors among MSM.

## Materials and Methods

### Design and Setting

This cross-sectional biological and behavioral survey took place in three major cities in the Republic of Panama—David, Panama, and Colon. Panama, a port city and the capital and home to 50 % of the total population of the country, is located south of the Panama Canal on the Pacific coast and is a high transit area due to tourism and commerce. Colon, also a port city, located on the north of the Panama Canal on the Atlantic coast, is the second most important economic center in the country because of the presence of the Duty Free Trade Zone. However, it has high unemployment and low income populations unlike other cities. David, the smallest of the three cities and representative of rural areas of the country, is the capital city of the province of Chiriqui, which borders Costa Rica on the country’s western border.

### Population

Respondent-driven sampling (RDS) methods 11 ^,^ 12 were used to recruit males who (1) self-identified as MSM (homosexual, bisexual, or transgender), (2) were 18 years and older, (3) had engaged in sex with another man or men in the last 12 months, and (4) who lived and/or worked in the survey city or in surrounding areas for at least 6 months and presented to the study site with a valid coupon. The RDS method was chosen as it allows recruitment of hidden populations such as MSM, drug users, and sex workers^11^^–^ 15 and has been used in 120 studies in more than 20 cities and with more than 32,000 individuals.^16^^,^ 17 The target sample size per city was determined by sample size calculations, estimates of MSM in each city reported by key informants, the extent of networking among this population, and budgetary and logistical feasibilities of recruitment of MSM. Target sample sizes of 300 in Panama and 200 each in David and Colon were calculated to estimate an HIV prevalence of 10 % with a precision of ±2.65 % and a minimum design effect of 1.5. The interviewers and “seeds” were selected before recruitment began. Additional seeds were chosen later to boost recruitment. Recruitment occurred from January 1 to October 14, 2011 in David, from January 28, 2011 to January 6, 2012 in Panama, and from July 5 to December 12, 2011 in Colon.

### Procedures

Several local organizations that work with MSM and transgendered persons provided valuable information about site locations, schedules, seed candidates, possible incentives, questionnaire validation and local population slang, and the selection of the personnel who work comfortably with MSM populations. Study procedures involved (1) interviews of candidate seeds and selection, (2) interview and blood collection from recruits.

Seeds were selected from candidates based on size of self-reported social and geographic networks of MSM, likelihood of referring three participants to the study, diversity of demographic characteristics, sexual identity, education level, employment status, nongovernmental organization/NGO membership, place of residence, and availability for participation in the study.

Coordinators at study sites screened recruits who presented with a valid coupon for eligibility and provided information about the study. Coordinators used a form to record responses to the eligibility criteria and their perceptions about the recruits’ sexual orientation. Each city had a different coupon code, thereby preventing participants in one city to be part of the survey in another. After recruits provided written informed consent, interviewers administered two questionnaires (one on risk assessment and another on social networks), provided pre- and posttest HIV counseling, performed a HIV rapid test via fingerstick, and collected a 10-mL blood sample. A recruit who met eligibility criteria and participated received three coupons and an incentive for participation. A participant also received an incentive for recruitment. Incentives included water bottles, baseball caps, and fanny packs. In Colon, additional incentives were provided in order to increase the recruitment rate. Male condoms and lubricants were always distributed in addition to incentives.

Prior to receiving test results, participants were offered posttest counseling for STIs, prevention education on the importance of correct and consistent use of condoms and lubricants, hepatitis B vaccination, the risk of intravenous drug use, and prompt treatment for syphilis. If applicable, participants were referred to health institutions for medical attention. Also at this visit, participants were asked to provide the age, relationship, and reason for rejection for potential recruits who chose not to participate in the study, i.e., rejected coupons.

### Ethical Considerations

The Institutional Review Boards of the Walter Reed Army Institute of Research (Washington, D.C., USA) and the National Bioethics Committee (Republic of Panama) reviewed and approved the study protocol. Written consent was obtained from all participants prior to enrollment in the study. Recruitment code numbers were used to track RDS recruitment, and names were not collected; the written consent form could be signed with a real or fictitious name.

### Laboratory Measures

Blood collection, transfer, testing, and laboratory assays used in this survey have been described previously in a survey of female sex workers.^18^ In brief, blood samples were evaluated for HIV [antibody to HIV (anti-HIV) and p24 antigen], antibody to hepatitis C virus (anti-HCV), hepatitis B surface antigen (HBsAg), antibody to hepatitis B core antigen (anti-HBc), antibody to herpes simplex type 2 virus (anti-HSV2), and syphilis. All serological tests, except HIV rapid tests, syphilis, and anti-HSV2 testing were conducted on an AxSYM (Abbott, Wiesbaden, Germany) using microparticle enzyme immunoassay (MEIA) technology and were repeated in duplicate if initial testing was reactive. Syphilis testing was performed using rapid plasma reagin (RPR) and anti-HSV2 using enzyme-linked immunoassay (ELISA). For HIV, samples were repeated in duplicate irrespective of the initial MEIA result.

Positive screening results were confirmed for HIV (Western Blot), HCV (recombinant immunoblot assay/RIBA), and syphilis (Treponema pallidum hemagglutination assay/TPHA). HIV infection and HCV seropositivity were defined as a repeat reactive MEIA confirmed by a positive Western blot for HIV or a positive RIBA for HCV. Participants who tested HBsAg positive and anti-HBc negative were considered HBV infected. Resolved HBV infection was defined as a positive anti-HBc and negative HBsAg. Active syphilis infection was defined as positive results for both RPR and TPHA assays.

### Data Analysis and Management

The recruiter-recruit relationship was tracked by using Coupon Manager V 3.3 software which facilitates tracking of recruitment, coupon numbers, and respondent compensation. Each coupon had its unique code that linked participants to their recruiters. The eligibility screening and/or interview questionnaires elicited information on age, education, employment status, income, other demographics, social networking, sexual orientation, sexual behavior, in the prior 2 months with each partner type and focused on number of sexual partners, regular female or male partnerships, preference of oral, vaginal or anal sex, insertive or receptive intercourse, condom use, drug, and alcohol use. Network size was determined by responses in increasing order of preference to three social network questions on the social network questionnaire (1) “How many MSM or trans persons do you know who live in this city?” (2) “How many of these persons you know are over the age of 18 years?” (3) “How many of these persons over 18 years have you seen or spoken to in the last 30 days?”

Unadjusted sample proportions and measures of central tendency for continuous variables were calculated excluding nonrandomly sampled seeds. We used respondent-driven sampling analysis tool (RDSAT) version 6.0 to generate adjusted population proportions and 95 % confidence intervals, to estimate within-group recruitment (or homophily), and to calculate the number of waves required to reach sample equilibrium for key variables reported here. RDSAT population estimates adjust for each participant’s network size and differential recruitment patterns or homophily. Questionnaire and laboratory results were double-data entered into a FileMaker Pro database. Responses to eligibility criteria captured by interviewers were double-data entered into Excel 2007 spreadsheets. All data management and unadjusted analyses were performed with SAS 9.2 (SAS, North Carolina, USA).

## Results

### Recruitment Characteristics

**FIG. 1:**
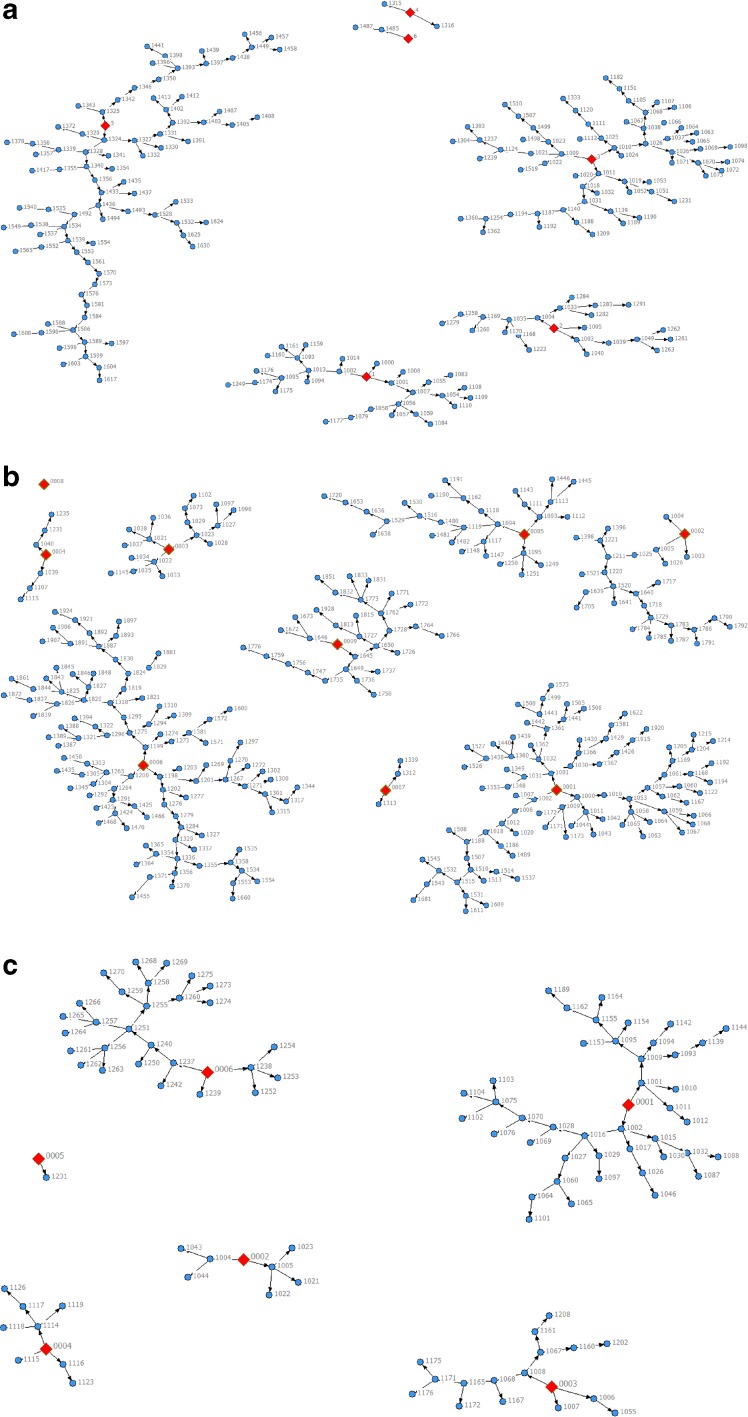
Recruitment chains among participants in David, Panama, and Colon. Nonrandomly sampled seeds are indicated as diamond shapes. Recruits are indicated as *circles*. **a** David. **b** Panama. **c** Colon.

Of the MSM recruited from three cities in Panama, the numbers of seeds and waves they generated varied by city. Six seeds in David, 9 seeds in Panama, and 6 seeds in Colon generated 22, 12, and 6 maximum waves, respectively (Fig. 1a–c); one seed in Panama was nonproductive. Seeds in Panama had the largest average network size (295.3, interquartile range/IQR 25.0–100.0) followed by Colon (46.7, IQR 20.0–80.0) while seeds in David had the smallest (21.7, IQR 12.0–30.0). Similar to seeds in Panama, recruits also had the largest network size (mean, IQR: Panama 48.8, 4.0–20.0; David 18.6, 5.0–20.0; Colon 17.3, 4.5–20.0). Thirty-three percent of recruits who were issued coupons participated in the survey. All variables of interest met equilibrium in all three cities except for sex worker status in Colon; seven waves were needed for equilibrium to be reached while only six waves were attained in the longest chain (Fig. 1c). The final unadjusted analyses excluded the nonrandomly sampled seeds and included 204 recruits from David, 306 from Panama, and 93 from Colon for a total of 603 participants. Five recruits who presented to the study site were excluded from analysis for not having a coupon. Recruits reported “interested in this subject” as the most common reason for enrolling in the study (David 66.0 %, Panama 62.0 %, Colon 68.0 %) followed by wanting to know their HIV status (David 22.0 %, Panama 36.0 %, Colon 20.0 %) and if “I have sexually transmitted disease” (David 39.0 %, Panama 31.0 %, Colon 19.0 %).

### RDS-Adjusted HIV, Syphilis, and Other Viral STI Prevalence

HIV prevalence varied by city, from the lowest of 6.6 % in David, 29.4 % in Panama to the highest of 32.6 % in Colon (Table 1). These differences were mirrored by active syphilis infection rates (David 16.0 %, Panama 24.7 %, Colon 31.6 %) and HSV-2 seropositivity rates (David 38.4 %, Panama 62.6 %, Colon 72.9 %) (Table 1). Hepatitis B infection prevalence was highest in Panama (3.4 %) whereas resolved HBV infection prevalence was highest in Colon (40.6 %) (Table 1). Only two recruits (0.8 %, 95 % C.I. 0.0–1.2 %) were HCV-seropositive and both were from Panama; RDSAT estimates could not be generated for David and Colon.

**TABLE 1:** Prevalence of HIV and other sexually transmitted infections among men who have sex with men from three cities, Panama, 2011–2012

Sexually transmitted infections	City											
	David, *N* = 204				Panama, *N* = 306				Colon, *N* = 93			
	*N*	Unadjusted prevalence %	Adjusted prevalence %	(95 % C.I.)	*N*	Unadjusted prevalence %	Adjusted prevalence %	(95 % C.I.)	*N*	Unadjusted prevalence %	Adjusted prevalence %	(95 % C.I.)
HIV												
Infected	16	8.0	6.6	(2.2–11.4)	76	25.0	29.4	(19.7–39.7)	32	34.0	32.6	(18.0–47.8)
Uninfected	187	92.0	93.4	(88.6–97.8)	230	75.0	70.6	(60.3–80.3)	59	63.0	67.4	(52.2–82.1)
Missing	1	0.0	–^a^	–^a^	0	0.0	–^a^	–^a^	2	2.0	–^a^	–^a^
Syphilis												
Infected	35	17.0	16.0	(8.9–24.2)	77	25.0	24.7	(16.7–32.9)	29	31.0	31.6	(14.8–47.5)
Uninfected	168	82.0	84.0	(75.8–91.1)	229	75.0	75.3	(67.1–83.3)	62	67.0	68.4	(52.5–85.2)
Missing	1	1.0	–^a^	–^a^	0	0.0	–^a^	–^a^	2	2.0	–^a^	–^a^
HSV-2												
Seropositive	74	36.0	38.4	(27.9–48.9)	182	59.0	62.6	(52.8–71.0)	63	68.0	72.9	(57.4–85.8)
Seronegative	129	63.0	61.6	(51.1–72.1)	123	40.0	37.4	(29.0–47.3)	28	30.0	27.1	(14.2–42.6)
Missing	1	0.5	–^a^	–^a^	1	0.3	–^a^	–^a^	2	2.0	–^a^	–^a^
HBV												
Infected	1	0.5	2.2	(0.0–4.7)	14	5.0	3.4	(1.3–6.3)	0	0.0	–^a^	–^a^
Resolved	19	9.0	10.0	(4.8–16.8)	86	28.0	29.4	(20.0–38.3)	30	32.0	40.6	(21.9–54.4)
Uninfected	183	90.0	87.8	(81.5–94.1)	206	67.0	67.3	(58.2–76.7)	61	66.0	59.4	(45.6–78.1)
Missing	1	0.5	–^a^	–^a^	0	0.0	–^a^	–^a^	2	2.0	–^a^	–^a^

*C.I.* confidence interval

^a^Indicates no results were generated

### RDS-Adjusted Sociodemographic and Behavioral Characteristics

A majority of MSM reported being single (76.8-87.6 %), employed (52.4-69.6 %), and of Panamanian nationality (96.8-99.9 %) (Table 2). Age, income, and education attained at the time of the survey differed by city (Table 2). Recruits from David were younger (median age 24 years, IQR 20–30 years), earned less (median income USD 300, IQR 75–416), and were more educated (over half had attended technical schools or university) whereas recruits from Colon were more similar to those from Panama in age (median age 26 years, Colon IQR 22–38, Panama IQR 22–32), income (median income (IQR), Colon USD 400 (250–700); Panama; USD 450 (IQR 300–700)), and education (attended/completed secondary school, Panama 55.6 %; Colon 53.4 %)(Table 2).

**TABLE 2:** Summary of sociodemographics and prevalence of sexual behaviors among men who have sex with men from three cities, Panama, 2011–2012

Characteristics	City											
	David, *N* = 204				Panama, *N* = 306				Colon, *N* = 93			
	*N*	Unadjusted prevalence %	Adjusted prevalence %	(95 % C.I.)	*N*	Unadjusted prevalence %	Adjusted prevalence %	(95 % C.I.)	*N*	Unadjusted prevalence %	Adjusted prevalence %	(95 % C.I.)
Sociodemographic												
Age												
24 or less	110	54.0	61.0	(47.8–69.3)	130	42.0	38.0	(28.2–47.4)	37	40.0	40.8	(23.8–62.7)
25–30	44	22.0	15.7	(9.5–24.9)	83	27.0	31.9	(23.1–41.0)	16	17.0	17.9	(7.9–34.0)
31+	49	24.0	23.4	(15.6–34.6)	92	30.0	30.1	(21.3–40.4)	38	41.0	41.3	(21.2–55.5)
Missing	1	0.0	–^d^	–^d^	1	1.0	–^d^	–^d^	2	2.0	–^d^	–^d^
Highest level of education												
Attended/completed primary	10	5.0	7.7	(2.3–13.7)	8	3.0	2.6	(0.3–6.6)	7	8.0	10.1	(1.5–21.6)
Attended/completed secondary	74	36.0	37.7	(28.1–51.1)	148	48.0	55.6	(46.1–65.1)	53	57.0	53.4	(37.6–70.9)
Technical/University	119	58.0	54.6	(41.7–65.7)	149	49.0	41.8	(32.4–51.9)	31	33.0	36.5	(20.9–53)
Missing	1	1.0	–^d^	–^d^	1	1.0	–^d^	–^d^	2	2.0	–^d^	–^d^
Marital status^a^												
Single	171	84.0	81.3	(71.9–89.6)	263	86.0	87.6	(80.7–93.2)	68	73.0	76.8	(60.2–89)
Nonsingle	32	16.0	18.7	(10.4–28.1)	41	13.0	12.4	(6.8–19.3)	19	20.0	23.2	(11.0–39.8)
Missing	1	0.0	–^d^	–^d^	2	1.0	–^d^	–^d^	6	6.0	–^d^	–^d^
Nationality^b^												
Foreign national	1	0.0	0.1	(0.0–0.1)	12	4.0	3.2	(1.0–6.2)	0	0.0	–	–
Panamanian	201	99.0	99.9	(99.9–100)	281	92.0	96.8	(93.8–99)	92	99.0	–	–
Missing	2	1.0	–^d^	–^d^	13	4.0	–^d^	–^d^	1	1.0	–^d^	–^d^
Employed currently												
Yes	119	58.0	60.5	(50.5–71.5)	213	69.0	69.6	(60.4–78.5)	45	48.0	52.4	(30.4–69.1)
No	84	41.0	39.5	(28.5–49.5)	89	29.0	30.4	(21.5–39.6)	44	47.0	47.6	(30.9–69.6)
Missing	1	1.0	–^d^	–^d^	4	2.0	–^d^	–^d^	4	4.0	–^d^	–^d^
Monthly income, USD												
400 or less	138	68.0	76.3	(64.8–84.7)	118	39.0	49.7	(36.0–60.1)	28	30.0	47.7	(19.2–67.0)
401–700	30	15.0	16.6	(8.6–27.8)	70	23.0	30.5	(21.1–42.5)	14	15.0	23.0	(6.8–44.9)
701+	20	10.0	7.1	(3.2–12.5)	62	20.0	19.9	(11.3–31.3)	13	14.0	29.3	(14.7–56.5)
Missing	16	8.0	–^d^	–^d^	56	18.0	–^d^	–^d^	38	41.0	–^d^	–^d^
Sexual risk behavior												
Sexual orientation												
Heterosexual	5	2.0	6.0	(0.6–12.0)	7	2.0	2.6	(1.0–5.7)	4	4.0	4.2	(0.6–9.9)
Homosexual	119	58.0	50.1	(40.2–63)	179	58.0	55.8	(46.5–66.6)	61	66.0	49.8	(35.4–68.0)
Transsexual/transvestite	9	4.0	2.4	(0.0–5.7)	45	15.0	11.2	(5.5–17.7)	6	6.0	6.1	(0.3–19.7)
Bisexual	66	32.0	41.5	(29.9–52.8)	58	19.0	30.4	(19.8–39.1)	20	22.0	39.8	(21.9–51.7)
Missing	5	2.0	–^d^	–^d^	17	6.0	–^d^	–^d^	2	2.0	–^d^	–^d^
Sexual preference												
Men only	140	69.0	60.2	(49.9–70.6)	259	85.0	80.2	(72.4–88.8)	67	72.0	60.1	(44.6–78.5)
Women only	11	5.0	10.4	(3.8–17.1)	8	3.0	5.7	(1.8–11.0)	2	2.0	10.1	(0.0–21.9)
Men and women	50	25.0	29.4	(20.8–39.3)	30	10.0	14.1	(6.7–20.4)	21	23.0	29.8	(14.5–48.7)
Missing	3	1.0	–^d^	–^d^	9	3.0	–^d^	–^d^	3	3.0	–^d^	–^d^
Type of regular partners, past 12 months												
All men	128	63.0	83.6	(69.7–90.4)	252	82.0	87.5	(80.8–94.4)	67	72.0	82.0	(69.4–94.4)
Men and women	25	12.0	16.4	(9.6–30.3)	20	7.0	12.5	(5.6–19.2)	10	11.0	18.0	(5.6–30.6)
Missing	51	25.0	–^d^	–^d^	34	11.0	–^d^	–^d^	16	17.0	–^d^	–^d^
Sex worker												
Yes	15	7.0	7.9	(2.4–15.9)	36	12.0	7.5	(3.4–13.0)	6	6.0	7.0	(0.0–23.1)
No	182	89.0	92.1	(84.1–97.6)	265	87.0	92.5	(87.0–96.6)	85	91.0	93.0	(76.9–100)
Missing	7	3.0	–^d^	–^d^	5	2.0	–^d^	–^d^	2	2.0	–^d^	–^d^
Partner HIV+												
Yes	2	1.0	–^d^	–^d^	15	5.0	2.4	(0.7–5.0)	7	8.0	9.4	(0.4–27.4)
No	101	50.0	61.2	(46.7–74.0)	133	43.0	53.0	(44.6–67.0)	54	58.0	69.6	(46.6–85.3)
Don’t Know	50	25.0	38.8	(26.0–53.3)	107	35.0	44.6	(30.8–52.7)	18	19.0	21.0	(8.4–40.7)
Missing	51	25.0	–^d^	–^d^	51	17.0	–^d^	–^d^	14	15.0	–^d^	–^d^
Number of sexual contacts, different people, past 2 months												
Two+	106	52.0	43.0	(32.4–53.4)	151	49.0	49.4	(40.4–59.8)	47	51.0	54.3	(39.0–72.3)
One	53	26.0	29.8	(20.1–39.5)	112	37.0	41.9	(31.5–51.1)	28	30.0	30.5	(15.4–46.1)
None	43	21.0	27.2	(18.4–38.0)	28	9.0	8.7	(4.6–13.5)	9	10.0	15.2	(3.2–29.3)
Missing	2	1.0	–^d^	–^d^	15	5.0	–^d^	–^d^	9	10.0	–^d^	–^d^
Type of sexual contact with men												
Anal receptive^c^	119	58.0	56.7	(46.2–66.3)	200	65.0	64.3	(55.3–72.0)	71	76.0	71.3	(55.2–82.5)
Anal insertive^c^	118	58.0	55.1	(45.5–65.5)	199	65.0	62.7	(53.2–71.0)	45	48.0	49.9	(35.3–67.4)
Condom use, anal sex, nonregular partner												
Never	11	5.0	9.0	(2.7–16.3)	25	8.0	10.2	(4.8–16.3)	7	8.0	5.9	(1.1–14.4)
Inconsistent	42	21.0	25.4	(15.1–36.2)	104	34.0	42.9	(30.1–50.4)	23	25.0	31.6	(14.7–47.6)
Always	130	64.0	55.0	(44.2–66.1)	130	42.0	44.2	(37.4–57.1)	46	49.0	46.1	(30.9–64.4)
I do not have anal sex	15	7.0	10.6	(4.7–19.0)	7	2.0	2.7	(0.6–5.5)	15	16.0	16.4	(4.4–31.3)
Missing	6	3.0	–^d^	–^d^	40	13.0	–^d^	–^d^	2	2.0	–^d^	–^d^
Sex with injecting user												
Yes	5	2.0	1.5	(0.1–3.9)	14	5.0	5.2	(1.4–10.4)	1	1.0	0.8	(0.0–2.9)
Don’t Know	20	10.0	7.8	(3.4–11.7)	43	14.0	13.7	(6.7–19.3)	4	4.0	4.3	(0.4–12.1)
No	177	87.0	90.8	(86.4–95.6)	209	68.0	81.2	(74.0–89.6)	83	89.0	94.9	(86.8–99.0)
Missing	2	1.0	–^d^	–^d^	40	13.0	–^d^	–^d^	5	5.0	–^d^	–^d^
Sexual contact with foreigners												
Yes	64	31.0	24.4	(17.3–32.5)	152	50.0	46.4	(36.7–56.4)	26	28.0	29.6	(17.6–45.0)
No	140	69.0	75.6	(67.5–82.7)	149	49.0	53.6	(43.6–63.3)	65	70.0	70.4	(55.0–82.4)
Don’t Know	0	0.0	–^d^	–^d^	1	0.0	–^d^	–^d^	0	0.0	–^d^	–^d^
Missing	0	0.0	–^d^	–^d^	4	1.0	–^d^	–^d^	2	2.0	–^d^	–^d^
Relationship to recruiter												
Acquaintance/close friend/sexual partner/relative	184	90.0	85.9	(75.4–95.3)	272	89.0	86.8	(79.9–93.7)	81	87.0	90.5	(81.7–96.8)
A stranger, someone you met for the first time	19	9.0	14.1	(4.7–24.6)	31	10.0	13.2	(6.3–20.1)	11	12.0	9.5	(3.2–18.3)
Missing	1	1.0	–^d^	–^d^	3	1.0	–^d^	–^d^	1	1.0	–^d^	–^d^

*C.I.* confidence interval

^a^Nonsingle marital status included participants who were married, or divorced, or widowed, or cohabiting

^b^RDSAT estimates could not be generated since recruitment was from a single group

^c^Statistics are not presented for those who did not check the response

^d^Indicates no results were generated

While a majority of participants from the three cities self-identified as homosexual (49.8–55.8 %), at least a third in each city self-identified as heterosexual or bisexual (33.0–47.5 %)(Table 2). At least one among five participants (19.8–39.9 %) reported a sexual preference for both men and women or women only (Table 2). In each city, a majority of participants reported having had two or more sexual contacts with different persons within 2 months of the interview (3 cities, median, 2 (IQR1-3)). Up to three-quarters of participants (56.7–71.3 %) reported engaging in receptive anal intercourse with men. Less than half of participants in Panama and Colon, and a little over half in David, reported always using a condom for anal sex with men who were not regular partners. Self-reported prior sexual contact with person(s) from other countries was highest in Panama (46.4 %).

## Discussion

We conducted a cross-sectional biobehavioral survey among MSM in the Republic of Panama and utilized respondent-driven sampling for recruitment in three cities over a span of approximately 6–12 months. Among the MSM in the three cities we surveyed who were young (median of 24–26 years), RDS-adjusted HIV, syphilis, and HSV-2 prevalences were high. We found HIV prevalence was 8–40 times higher than the 2011 overall estimate of 0.8 % among the general population in Panama aged 15–49 years, and considerably higher than an overall estimate of 8.9 % reported by the last study conducted among MSM in the cities of Panama and Colon, which used convenience sampling.^9^ Syphilis infection rates, ranging from 16–32 %, were at least 16 times higher than the prevalence of approximately 1 % reported among MSM in the last study. A survey among another high risk group—female sex workers (FSWs) who were recruited by venue-based, time-space sampling in the three cities—revealed an HIV prevalence of 2–4.2 %.^9^^,^ 18 HSV-2 seropositivity rates in Panama (62.6 %) and Colon (72.9 %) were higher than 44.3 % reported previously among MSM by Soto et al. Our survey among FSWs sampled from Panama and Colon in 2009–2011 revealed HSV-2 seropositivity rates of 71.2 and 76.7 %, respectively.^18^

Although estimates in populations generated by methodologically different surveys may not be comparable due to varying precision, HIV prevalence among MSM in two of the three Panamanian cities we studied ranked highest when compared to estimates from reporting countries in the Western hemisphere cited in the UNAIDS 2012 Global Report^8^; prevalence in Central American cities ranged from approximately 6–17 % and in South American cities from 9–20 %. In 2012, the highest prevalence among MSM in the range of 31–37 % was reported among Central African countries. Compared to other countries in the world, Panama is reported to provide good treatment coverage and moderately high levels of testing among MSM; of those eligible for treatment, 40–59 % were reported to have been receiving antiretroviral treatment at the end of 2011 and level of HIV testing among MSM was 50–74 % in 2011, a level comparable to the United States.^8^ However, given the top reasons for participation in the survey, it is probable participants did not know their status. The high HIV rates we observed indicate young sexually active MSM populations in the three Panamanian cities continue to experience concentrated epidemics, defined as a prevalence >5 % in a high risk subpopulation and <1 % in the general population.^19^

Targeting HIV preventive efforts to high risk subpopulations is recommended for concentrated epidemics.^10^ An understanding of the drivers of ongoing HIV transmission is necessary to design effective prevention programs. In this survey, 33–47 % who participated self-identified as heterosexual or bisexual despite having met the eligibility criteria of having had sex with a male within the previous 12 months. Other studies among MSM in the region have reported similar findings.^20^^,^ 21 Creswell et al. in 2012 reported over 40 % of MSM self-identified as heterosexual or bisexual in a RDS study reporting adjusted HIV prevalences of 8.8 and 10.8 % in two cities of El Salvador.^20^ Tabet el al. found 29.2 % of MSM in Peru self-identified as heterosexual or bisexual in a cross-sectional study which used snowball sampling for recruitment.^21^ It is possible our findings reflect the discrimination MSM experience which may be associated with HIV transmission. Homophobia has been recognized as a driver of the HIV epidemic among MSM in that it prevents men from accessing HIV prevention programs such as HIV and STI testing, counseling, and free condom, mental health, and education services.^22^ Future studies clearly are needed in order to define whether or not this is a specific driver of the epidemic in Panama.

At most, 55 % reported always using a condom for anal sex with nonregular partners. While Panama places in the upper third quartile for condom use among MSM among reporting countries in the world,^8^ higher condom use among young MSM should remain a target for prevention programs to interrupt further secondary transmission of HIV and STI among this population and to prevent transmission to women. At least one in five participants reported having sex with men and women or women only. Furthermore, results of this survey indicate bridging to MSM from other countries; almost a quarter to half of participants in the three cities reported prior sexual contact with a person or persons from other countries. Phylogenetic analysis of HIV subtypes isolated from MSM and other risk groups recruited in prior and ongoing biobehavioral surveys may provide an understanding of the extent of connections within and between groups. However, prevention programs should continue to target increased HIV testing and more condom use among young MSM and their partners to decrease secondary transmission and decrease the potential for HIV transmission from MSM to other populations.

There were limitations to this survey. We did not elicit information as to whether participants who tested HIV positive in the survey had known their status nor did we estimate whether infections diagnosed in the survey were prevalent or incident. Consequently, it is difficult to determine whether high prevalence of HIV infection we observed were due to a high incidence from transmission among MSM who were unaware of their infection or good treatment coverage of HIV-positive individuals who were aware of their infection status and had been linked to care before participation in the survey. MSM who participated in this survey were young and sexually active. Our results may not be representative of older MSM who were not recruited by RDS perhaps due to factors such as a reluctance to disclose their behavior possibly from the prevalent stigma and discrimination against MSM in Panama,^5^ knowing their HIV status, or being more established economically which precluded having time to participate in the survey, or being attracted by remuneration for participation. While RDS has been used widely for reasons of being an effective data collection method for inaccessible populations and being logistically easy to implement, estimates and confidence intervals generated from RDS have received criticism for not being representative of the target population and for being insufficiently precise.^23^

Our findings reveal several potential targets for preventive intervention programs. Prevalence of resolved or natural immunity to hepatitis B was comparatively lower in Panama and David and more than half of the participants we surveyed were HBV uninfected. These rates indicate an opportunity for intervention with HBV vaccination among MSM in these cities who are generally at higher risk of infection. A majority of participants reported interest in the survey as the primary reason for participation followed by HIV testing and wanting to know their STI status. Other preventive measures include increased sexual health programs for MSM which include HIV and STI testing and counseling, condom distribution, and risk reduction.

## Conclusions

HIV prevalence was high among young sexually active MSM we surveyed in three cities in Panama. The HIV and syphilis epidemics are concentrated in men who have sex with men in these cities. Prevention programs should target increased testing and treatment for HIV and STI, condom awareness and use among MSM.
